# Prevalence of African Horse Sickness Virus Antibodies in Horses and Selected Wildlife in Four Geographical Regions of Nigeria

**DOI:** 10.1155/vmi/4106678

**Published:** 2025-08-21

**Authors:** C. N. Chinyere, A. C. Ajaebili, I. K. Peter-Ajuzie, H. B. Galadima, O. B. Daodu, O. I. Fatola, C. C. Okolo, B. A. Alaba, O. O. Akinniyi, D. O. Omoniwa, E. R. Edeh, A. B. Olorunfemi, T. A. Olayinka, O. Ojurongbe, D. O. Oluwayelu, A. B. Muhammad, M. B. Abubakar, C. A. Meseko, A. N. Happi, C. T. Happi, A. S. Bakarey, M. H. Groschup, J. O. Olopade

**Affiliations:** ^1^Department of Veterinary Microbiology, University of Ibadan, Ibadan, Oyo, Nigeria; ^2^Humboldt Research Hub for Zoonotic Arboviral Diseases, University of Ibadan, Ibadan, Oyo, Nigeria; ^3^Regional Centre for Animal Influenza and Other Transboundary Animal Diseases, National Veterinary Research Institute, Vom, Jos, Plateau, Nigeria; ^4^Department of Veterinary Anatomy, University of Ibadan, Ibadan, Oyo, Nigeria; ^5^Department of Veterinary Anatomy, University of Nigeria, Nsukka, Enugu, Nigeria; ^6^Department of Veterinary Medicine, University of Maiduguri, Maiduguri, Borno, Nigeria; ^7^Department of Veterinary Microbiology, University of Ilorin, Ilorin, Kwara, Nigeria; ^8^Department of Veterinary Medicine, University of Nigeria, Nsukka, Enugu, Nigeria; ^9^Department of Veterinary Medicine, University of Ibadan, Ibadan, Oyo, Nigeria; ^10^Department of Veterinary Medicine, Surgery and Radiology, University of Jos, Jos, Plateau, Nigeria; ^11^Department of Medical Microbiology and Parasitology, Ladoke Akintola University of Technology, Ogbomosho, Oyo, Nigeria; ^12^Humboldt Research Hub-Centre for Emerging and Re-Emerging Infectious Diseases, Ladoke Akintola University of Technology, Ogbomosho, Oyo, Nigeria; ^13^BlueBlood Veterinary Clinic, Federal Capital Territory, Abuja, Nigeria; ^14^Department of Veterinary Microbiology, University of Maiduguri, Maiduguri, Borno, Nigeria; ^15^African Centre of Excellence for Genomics of Infectious Diseases, Redeemer's University, Ede, Osun, Nigeria; ^16^Institute of Medical Research and Training, College of Medicine, University of Ibadan, Ibadan, Oyo, Nigeria; ^17^Institute for Novel and Emerging Infectious Diseases, Friedrich Loeffler Institute, Insel Riems, Germany

**Keywords:** African horse sickness, antibodies, equine, Nigeria, wildlife

## Abstract

African horse sickness (AHS) is a severe, infectious arthropod-borne disease of equids caused by the AHS virus (AHSV). It is endemic in Sub-Saharan Africa, and several sporadic outbreaks of the disease have been reported in Nigeria in the past 5 decades. Following a recent outbreak of the disease in Lagos State, this study was conducted to investigate the prevalence of (AHSV) antibodies in apparently healthy horses and some selected wildlife sampled in four geographical regions of Nigeria. Using a competitive enzyme-linked immunosorbent assay, 575 serum samples collected from horses in five locations, namely Abuja (*n* = 220), Enugu (*n* = 69), Oyo (*n* = 64), Plateau (*n* = 145) and Yobe (*n* = 77), were screened for anti-AHSV antibodies. In addition, we screened 134 wildlife, consisting of rodents, bats, and birds. The results obtained revealed an overall seroprevalence rate of 89.9% (*n* = 517) in horses, with the highest (100%) and lowest (75%) recorded in Enugu State, southeastern Nigeria, and Oyo State, southwestern Nigeria, respectively. There was a 0% prevalence amongst the wildlife examined. This high seroprevalence rate in horses shows that AHS is widespread among the horse population in different regions of Nigeria, suggesting significant exposure to the virus. In addition, the high AHS seroprevalence suggests endemicity of the disease in Nigeria, which could be attributed to *Culicoides* vector activities. Although we could not distinguish between antibodies due to natural infection and those induced by vaccination, our findings emphasize the need for continuous surveillance of AHS in horses in Nigeria to track the possible evolution of the virus in the country and aid the formulation of effective prevention and control strategies against the disease.

## 1. Introduction

African horse sickness (AHS) is a viral disease of equids that is transmitted by hematophagous insects and endemic to Sub-Saharan African countries [1, 2]. The disease is caused by the AHS virus (AHSV) (*Orbivirus alphaequi*), which is a segmented, nonenveloped dsRNA virus with a diameter of 55–70 nm, belonging to the genus *Orbivirus* in the family Sedoreoviridae and order Reovirales [3]. The viral genome is approximately 18.5 kb in length and composed of 10 segments of dsRNA enclosed within a triple-layer capsid; it encodes seven structural (VP1–VP7) and four nonstructural proteins (NSI, NS2, NS3, and NS3a) [4, 5]. There are nine antigenically distinct serotypes (AHSV-1 to AHSV-9) identified by the virus neutralization test [6].

AHS is listed as a notifiable disease by the World Organization for Animal Health because of its severity and potential for rapid global spread [7]. Severe and often, disease with a mortality rate above 94% is seen frequently in horses, while zebras, mules, and African donkeys are asymptomatic reservoirs that play a key role in the disease's epidemiology [7–10]. In addition, four clinical forms of the disease, horse sickness fever, mixed, cardiac and pulmonary, have been described [7]. Biting midges (*Culicoides* spp.) are the primary vectors of AHSV, with *C. imicola* being the most significant species for transmission [11], although *C. Bolitinos* also plays an important role [12]. Also, AHSV has been isolated from the dog *Rhipicephalus sanguineus* [13] and *Hyalomma dromedarii* [14]. However, ticks and mosquitoes do not play an important role in the epidemiology of AHS [15]. Apart from dogs, Hanekom et al. [16], seropositivity to AHS has been reported in some wild carnivores, goat, sheep, and elephant [17]. Severe epizootics of AHS have been reported in various African and Mediterranean countries [18]. The disease is endemic in Africa, with the most affected countries being in the Sub-Saharan region, particularly South Africa, where all nine serotypes of AHSV have been identified [7, 18, 19]. According to Baylis et al. [20], epidemic waves of AHS associated with climatic conditions appear every 10–15 years in South Africa. Senegal reported an outbreak of AHS in 2007, which was caused by AHSV Serotype 2 [21]; while another AHS outbreak occurred in April 2019, in Chad, causing a fatality rate of 85.11% [15]. Outside Africa, AHS outbreaks have been documented in the Middle East (1959–1963), Spain (Serotype 9 in 1966; Serotype 4 in 1987–1990), and Portugal (Serotype 4 in 1989) [22]. From 1959 to 1961, the disease spread as far as Pakistan and India, causing fatalities of approximately 300,000 equids [2, 23], while Thailand recorded an outbreak in February 2020 [24].

In Nigeria, between 1931 and 1967, AHS cases have been described only by clinical diagnosis and serological tests in the northern part of the country [25]. However, since the first known outbreak and subsequent detection of the virus from a dead horse in Nigeria in 1970 [26], sporadic outbreaks of AHS have occurred in different locations in the country [27, 28]. Unlike previous outbreaks in which AHSV Serotype 9 was confirmed, Fasina et al. [29] reported the first detection of a Serotype 2 AHSV in the northern hemisphere from an outbreak in Lagos in 2007. Furthermore, using real-time reverse transcription–polymerase chain reaction, Lazarus et al. [30] reported the detection of AHSV in tissue samples from a captive zebra that died in a Game Reserve in Bauchi State, Nigeria. Other studies by Baba et al. [31], Oladosu et al. [32], and Ehizibolo et al. [33] confirmed the detection of AHSV antibodies against Serotypes 4 and 9 in horses, donkeys, camels, and dogs in Nigeria, where only Serotypes 2, 4, and 9 have been confirmed. There is a dearth of data on the seroprevalence of AHSV antibodies amongst unusual host species in Nigeria.

The population of horses in Nigeria is believed to be over 1.2 million [34] and comprises of local (Arewa) breed and exotic (Argentine and Sudanese) breeds that are kept under an intensive management system for racing and polo games. Recently, an outbreak of AHS was reported in Lagos State in December, 2022, which was confirmed by real-time RT-PCR at the National Veterinary Research Institute, Vom [35]. Considering the recurrent epizootics of AHS in Nigeria, there is a need for continuous surveillance of the disease in the equine industry to forestall future outbreaks amongst unusual hosts to better understand the disease epidemiology. This study was therefore conducted to investigate the prevalence of antibodies against AHSV in horses and selected wildlife located in four geographical regions of Nigeria.

## 2. Methods

### 2.1. Study Location

**Figure 1:**
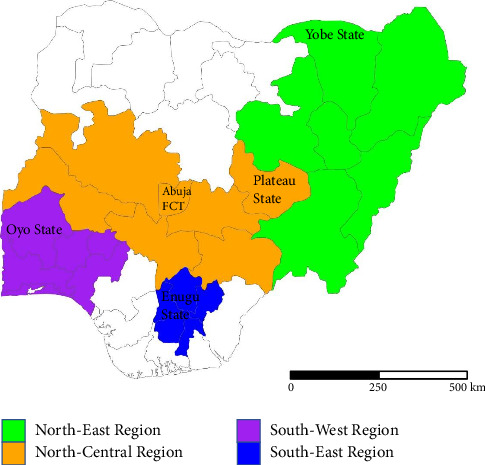
Map of Nigeria, showing locations of sample collection.

The study was carried out on horses at polo clubs and private stables spread across Nigeria (Figure 1). Specifically, study areas included North-Central (Abuja and Jos, Plateau State), northeastern (Damaturu, Yobe State), southeastern (Nsukka, Enugu State), and southwestern (Ibadan, Oyo State) regions of Nigeria. These horses were primarily kept for special events and ceremonial activities such as racing, polo games, and Durbar festivals. Selected wildlife including African giant rats (AGRs), bats, cattle egrets, crows, hedgehogs, pigeons, and squirrels had also been collected across these locations.

### 2.2. Ethical Considerations and Consent

Ethical clearance for this study was obtained from the Animal Care and Use Research Ethics Committee (ACUREC) of the University of Ibadan (UI-ACUREC/036-0224/23) for the equine study and from the National Veterinary Research Institute for the wildlife surveillance study. Verbal consent to participate was obtained from the horse owners after explaining the objectives of the study to them. Only horses of owners who consented were included in the study.

### 2.3. Study Design

The study was designed as a cross-sectional survey of apparently healthy exotic and indigenous horses in North-Central, northeastern, southeastern, and southwestern regions of Nigeria. The sampling method used was random sampling. Some selected species of wild animals such as pigeons, cattle egrets, AGRs, bats, squirrels, hedgehogs, and pied crow were also included in the study.

### 2.4. Sample Size Determination

The minimum sample size for horses was calculated using the formula outlined by Thrusfield [36]. A prevalence of 86.6% was used according to the result obtained by Ehizibolo et al. [33], while the confidence interval was set at 95% and the precision at 5%. An estimated 178 samples were obtained, but in order to increase the accuracy for different regions of the country, 575 horses were sampled.

### 2.5. Collection of Blood Samples

Standard physical restraint was first ensured to prevent injury and afford minimal pain elicitation during sampling. Using sterile syringes and needles, about 5–10 mL of blood was collected aseptically from each horse through the jugular vein. The blood samples were dispensed into appropriately labeled sterile plain bottles and transported in a cold box to the laboratory where they were allowed to clot. They were then centrifuged at 1500 g for 10 min, and the separated sera were kept at −20°C until analyzed for AHSV-specific IgG antibodies. For the wildlife, aliquots of serum samples stored from an ongoing HRH-ZAD project were obtained and analyzed as performed for the equine samples.

### 2.6. AHSV IgG Antibody Detection

The AsurDx antibody enzyme-linked immunosorbent assay (ELISA) test kit (BioStone Animal Health, Texas, USA) was used for the detection of AHSV IgG antibodies in horses according to the manufacturer's instructions. In brief, 90 μL of the AHSV diluent was added to each well of the antigen-coated plate. Aliquots (10 μL) of AHSV positive and negative control sera were added to two wells each of the antigen-coated plate, that is, Wells A1 and B1 and C1 and D1, respectively. 10 μL of each diluted (1:20 dilution) serum samples were added per well. The plate was gently rocked manually for 1 min to mix the contents and incubated for 30 min at room temperature. Thereafter, the solution in the wells was discarded, and the wells were rinsed 5 times with the wash buffer and then blotted dry on paper towels. 100 μL of horseradish peroxidase–conjugated antibody solution was added to each well of the plate and incubated for 30 min at room temperature. After incubation, the solution in the wells was discarded, and the plate was washed 5 times with wash buffer and blotted dry on paper towels. 100 μL of the TMB substrate solution was added to each well and incubated for 15 min at room temperature. After incubation, 100 μL of the stop solution was added to each well to stop the reaction, and thereafter, the plate was read in a plate reader at 450 nm wavelength. The optical density (OD) of the wells was measured at 450 nm within 15 min after color development had stopped. For the test to be valid, the percentage positive (PP) for the negative control must be < 40%, while the mean OD of the value of the positive control must be ≥ 0.5.

Note: Samples with *PP* values < 40% were considered negative, while those with PP values ≥ 40% were concluded to be positive.

### 2.7. Data Analysis

The data obtained were entered into Microsoft Excel spreadsheets and analyzed using the Statistical Package for the Social Sciences software (SPSS) (Version 20.0). The univariate analysis using Fisher's exact test was applied to test for the association between AHSV IgG prevalence and variables such as location, sex, and breed of horses. Odds ratio and 95% confidence interval were used to determine the strength of association between the results obtained and the variables. For the statistical analysis, *p* values < 0.05 were considered significant.

## 3. Results

The result revealed an overall prevalence of 89.9% (517/575) (Table 1). Based on location, all horses sampled in Enugu (southeastern region) were exposed to AHSV (100.0%, 69/69), followed by Abuja (95.0%, 209/220), Plateau (86.9%, 126/145), Yobe (84.4%, 65/77), and Oyo (75.0%, 48/64) (Table 1). Also, AHSV IgG prevalence was found to be higher in the female horses (90.4%, 357/395) than in the males (88.9%, 160/180) (Table 1). Furthermore, based on breed distribution, the Argentine breed of horse had the highest AHSV IgG prevalence (93.3%, 195/209) followed by the Talon breed (88.9%, 8/9), the Arewa breed (85.7%, 6/7) and the Sudanese breed (85.5%, 171/200) (Table 1). Based on some selected species of wild animals, the overall seroprevalence is 0% (0/134) (Table 2).

## 4. Discussion

The overall seroprevalence of AHSV in horses in the study areas was found to be 89.9% (517/575). This was higher than 86.6% previously reported in Nigeria [33]. Also, the prevalence in this study was higher than 46% in Ethiopia [37], 81% in Gambia [38], and 58.93% in Cameroon [39] but lower than 91.81% in Senegal [40]. The results according to earlier serological investigations conducted in Nigeria, 80.8% by Nawathe et al. [41], 89.7% by Oladosu et al. [32], and 84.3% by Adeyefa and Hamblin [42], show that this high prevalence in Nigeria has been consistent for over 3 decades.

**Figure 2:**
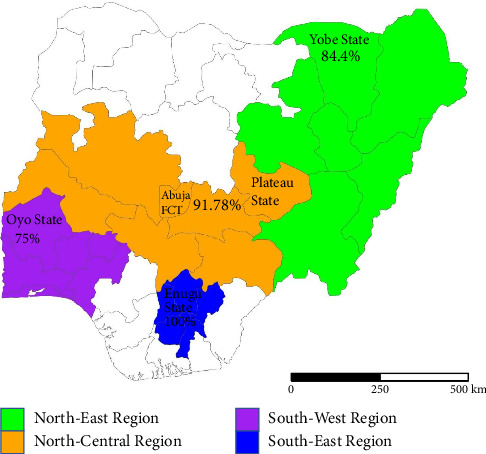
Geographical distribution of AHSV IgG among horses across regions in Nigeria.

The analysis indicated a significant difference in AHSV seroprevalence in the southeastern region compared to each of the North-Central, North-East, and South-West regions of Nigeria (*p* ≤ 0.0005; Table 1). The highest seroprevalence recorded in the South-East region (Table 1 and Figure 2) indicates the high level of exposure in the surveyed population, which reveals the endemicity of the disease in Nigeria, and further suggests a high rate of transmission, which could have resulted from the major *Culicoides* vector across the country.

Although there is no significant difference in AHSV seroprevalence based on sex, the analysis revealed that female horses were 1.2 times more likely to be exposed to AHSV than males (Table 1). This also indicates that there is no sex predisposition to the occurrence of AHS. This finding is consistent with the report of Kassa [43], who found that there was no significant variation in the seropositivity of male and female horses, which shows that both sexes can equally be affected by AHS.

Based on the breeds of the horse, exposure to AHSV among the Argentine breed was significantly higher than that observed among the Sudanese breed (*p*=0.0148; Table 1). This shows that the Argentine horses are more at risk compared to the Sudanese breed, and this could be associated with the susceptibility of the breed, especially when exposed in an endemic region. The study further showed that the Argentine breeds were 2.4 times more likely to be exposed to the virus than the Sudanese breed. In addition, the Talon horses were 1.4 times more likely to be exposed to AHSV compared to the Sudanese and Arewa (indigenous) breeds. This indicates that the exotic breeds of horses are more at risk compared to the Sudanese and Arewa (indigenous) breeds. Usually, they are highly susceptible to bites of *Culicoides* vectors and subsequent development of AHSV infection. In contrast, the indigenous breeds of horses are relatively resistant to bites of the insect vectors; hence, they are at lower risk for the disease compared to the exotic (cross) breeds [44]. Other factors that could lead to high seropositivity in some breeds could also be attributed to environmental conditions, management practices, geographical distributions, activity, and use (i.e., more often used for competitions or extensive riding).

The absence of seropositivity in some of the selected species of wild animals in Table 2 suggests that they might be refractory for AHSV, and that they are unlikely to act as reservoir or amplifiers of AHSV in the ecosystem, which indicates for now that only specific species, primarily equids, play a significant role in the epidemiology of the disease [17].

In Nigeria, some limitations which could lead to high prevalence of AHSV are a lack of vaccination, limited surveillance and monitoring, porous borders and movement of animals, and a lack of awareness and education. The high seroprevalence of AHS in horses in this study shows that the disease is widespread in Nigeria, suggesting its endemicity, which could be attributed to increased C*ulicoides* activities at varying levels of exposure. Also, this finding revealed that AHS can occur following long and short rainy seasons with an increase in the population of *Culicoides* midges and the movement of horses from one region to another. Mulualem et al. [45] had reported that the multiplications of *Culicoides* midges are influenced by ambient temperatures and prevailing weather conditions.

## 5. Conclusion

Our present findings revealed that AHS is highly prevalent in horses in Nigeria, which indicates that the disease remains a potent threat to the Nigerian equine population. Since there is no routine preventive vaccination against AHS in Nigeria, annual vaccination of both local and exotic horses is recommended; also, integrated vector control and good stable management practices are advocated to minimize the incidence of the disease. Suspected outbreaks in different regions of the country should be reported to the appropriate authorities for prompt investigation. This would help ascertain the circulating AHSV serotypes and identify the prevalent *Culicoides* species and other potential vectors involved. Continuous surveillance for AHS in the Nigerian equine populations and other species to track the possible evolution of the virus in the country is also advocated.

## Figures and Tables

**Table 1:** Risk factors associated with AHSV seroprevalence in Nigeria.

Features	Freq.	AHSV positive (%)	OR (95% CI)	*p* value
Locations (region)				
Enugu (South-East)	69	69 (100.0)	1	
Abuja (Federal Capital Territory)	220	209 (95.0)	0.1 (0.0–2.3)^a^	0.0719
Plateau (North-Central)	145	126 (86.9)	0.5 (0.0–0.8)^a^	0.0005^∗^
Yobe (North-East)	77	65 (84.4)	0.0 (0.0–0.7)^a^	0.0004^∗^
Oyo (South-West)	64	48 (75.0)	0.0 (0.0–0.4)^a^	< 0.0001^∗^
Sex				
Female	395	357 (90.4)	1.2 (0.7–2.1)	0.6543
Male	180	160 (88.9)	1	
Breed				
Argentine	209	195 (93.3)	2.4 (1.2–4.6)	0.0148^∗^
Talon	9	8 (88.9)	1.4 (0.2–11.3)	1.0000
Sudanese	200	171 (85.5)	1	
Arewa	7	6 (85.7)	1.0 (0.1–8.8)	1.0000
Unknown	150	137 (91.3)	1.8 (0.9–3.6)	0.1339
Total	575	517 (89.9)		

*Note:* Freq. = frequency.

Abbreviations: AHSV = African horse sickness virus; OR = odds ratio.

^a^Odds ratio was calculated by adding 0.5 to each value.

^∗^Significant association.

**Table 2:** Seroprevalence of AHS in some selected species of wild animals.

Species of animals	No. of animals tested	AHSV positive (%)
African giant rats	20	0 (0.0)
Bats	10	0 (0.0)
Cattle egrets	30	0 (0.0)
Crows	12	0 (0.0)
Hedgehogs	14	0 (0.0)
Pigeons	28	0 (0.0)
Squirrels	20	0 (0.0)
Total	134	0 (0.0)

## Data Availability

The data that support the findings of this study are available on request from the corresponding author. The data are not publicly available due to privacy or ethical restrictions.
